# *BTG1* Expression Correlates with the Pathogenesis and Progression of Ovarian Carcinomas

**DOI:** 10.3390/ijms141019670

**Published:** 2013-09-27

**Authors:** Yang Zhao, Wen-Feng Gou, Shuo Chen, Yasuo Takano, Yin-Ling Xiu, Hua-Chuan Zheng

**Affiliations:** 1 Department of Gynecology, the First Affiliated Hospital of China Medical University, Shenyang 110001, China; E-Mails: yida.zhaoyang@163.com (Y.Z.); chenshuo077003@163.com (S.C.); xiu561480485@163.com (Y.-L.X.); 2 Department of Biochemistry and Molecular Biology, Institute of Pathology and Pathophysiology, College of Basic Medicine, China Medical University, Shenyang 110001, China; E-Mail: xiaogouaeiou@163.com; 3 Clinical Cancer Institute, Kanagawa Cancer Center, Yokohama 241-0815, Japan; E-Mail: ytakano@gancen.asahi.yokohama.jp

**Keywords:** ovarian carcinoma, *BTG1*, phenotypes, tumorigenesis, progression

## Abstract

*BTG* (B-cell translocation gene) can inhibit cell proliferation, metastasis, and angiogenesis and regulate cell cycle progression and differentiation in a variety of cell types. We aimed to clarify the role of *BTG1* in ovarian carcinogenesis and progression. A *BTG1*-expressing plasmid was transfected into ovarian carcinoma cells and their phenotypes and related proteins were examined. *BTG1* mRNA expression was detected in ovarian normal tissue (*n* = 17), ovarian benign tumors (*n* = 12), and ovarian carcinoma (*n* = 64) using real-time RT-PCR. Ectopic *BTG1* expression resulted in lower growth rate, high cisplatin sensitivity, G_1_ arrest, apoptosis, and decreased migration and invasion. Phosphoinositide 3-kinase, protein kinase B, Bcl-xL, survivin, vascular endothelial growth factor, and matrix metalloproteinase-2 mRNA and protein expression was reduced in transfectants as compared to control cells. There was higher expression of *BTG1* mRNA in normal tissue than in carcinoma tissue (*p* = 0.001) and in benign tumors than in carcinoma tissue (*p* = 0.027). *BTG1* mRNA expression in International Federation of Gynecology and Obstetrics (FIGO) stage I/II ovarian carcinomas was higher than that in FIGO stage III/IV ovarian carcinomas (*p* = 0.038). Altered *BTG1* expression might play a role in the pathogenesis and progression of ovarian carcinoma by modulating proliferation, migration, invasion, the cell cycle, and apoptosis.

## 1. Introduction

Ovarian cancer is a malignant disease that poses a serious threat to women’s health; it is the fifth leading cause of cancer-related death in women. More than 90% of ovarian cancers are classified as epithelial and are believed to arise from the ovarian epithelium due to risk factors such as family history of ovarian carcinoma, and mutation. Ovarian cancer is disproportionately deadly because the lack of a sophisticated approach to early diagnosis means that most ovarian cancers are diagnosed at advanced stages, resulting in the five-year survival rate of ovarian cancer being 47% [1,2]. Tumorigenesis and progression of ovarian carcinoma is a multistage process, and increased understanding of the changes that occur in gene expression during carcinogenesis may result in the improvement of its diagnosis, treatment, and prevention.

The human B-cell translocation gene (*BTG*) family comprises six members (*BTG1*, *BTG2/TIS21/PC3*, *BTG3*, *BTG4/PC3B*, *Transducer of ErbB-2*, and *TOB2*) whose encoding proteins inhibit proliferation and regulate cell cycle progression and differentiation in a variety of cell types. BTG is a nuclear protein that is imported into the nucleus through a nuclear localization signal; its nucleocytoplasmic translocation depends on the stage of cell growth as mediated by a nuclear export signal [3–5]. *BTG1* was originally identified in a B-cell chronic lymphocytic leukemia at a t(q24;q22) translocation and was used as an indicator biomarker for complete remission of acute myeloid leukemia [6–8]. Human *BTG1* is localized on chromosome 12q22 and its 4704-nucleotide cDNA encodes 171 amino acids and 19 kDa protein [9]. The *N*-terminal domain of *BTG1* bears an LXXLL motif favoring nuclear accumulation, and another region encompassing Box A inhibiting nuclear localization [5]. The *C*-terminal domain of

*BTG1* is involved in interaction with the nuclear receptor TRα and the myogenic factor MyoD [10]. *BTG1* expression is highest in the G_0_/G_1_ phases of the cell cycle and is decreased when cells progress through G_1_. It is believed to be a potential tumor suppressor due to its inhibitory effects on proliferation and cell cycle progression [11]. Additionally, *BTG1* can bind to protein arginine methyltransferase (PRMT) 1 via the box C region [6,12]. *BTG1* expression in primary mouse bone marrow cells suppressed the outgrowth of erythroid colonies, which requires a *BTG1* domain that binds to PRMT1 [13]. Human carbon catabolite repressor protein-associated factor 1 (hCAF-1) can form a hCAF-1/BTG1 complex [14], which is dependent on the phosphorylation of a putative p34CDC2/cyclin E and p34CDK2/cyclin A kinase site on BTG1 Ser-159 [15]. *BTG1* is a Bcl-2-regulated mediator of apoptosis and contributes to antisense Bcl-2-mediated cytotoxic effects in breast cancer cells [16]. *BTG1* enhanced homeobox B9-mediated transcription in transfected cells and mediated their antiproliferative function [17]. *BTG1* overexpression induced increased apoptosis of NIH 3T3 cells, indicative of its pro-apoptotic effect [18]. *BTG1* overexpression may inhibit myoblast proliferation and stimulate terminal differentiation [19]. In macrophages, activator protein-1 and nuclear factor κB inhibition mediated by *BTG1* activation reversed the oxidative stress of the inducible nitric oxide synthase and cytokine genes [20].

As shown by DNA fragmentation and nuclear condensation, *BTG1* localizes to specific macrophage-rich regions in human lesions and apoptotic cells. *BTG1* mRNA is abundantly expressed in quiescent endothelial cells and decreased by the addition of angiogenic growth factors [17]. In this study, we describe the effects of *BTG1* overexpression on the phenotypes and related proteins of ovarian carcinoma cells. We examined *BTG1* mRNA expression in ovarian normal tissue, benign tumors, and carcinomas and compared it with clinicopathological parameters to clarify the roles of *BTG1* in ovarian carcinogenesis and subsequent progression.

## 2. Results

### 2.1. Effects of *BTG1* Overexpression on the Phenotypes and Related Proteins of Ovarian Carcinoma

**Figure 1:**
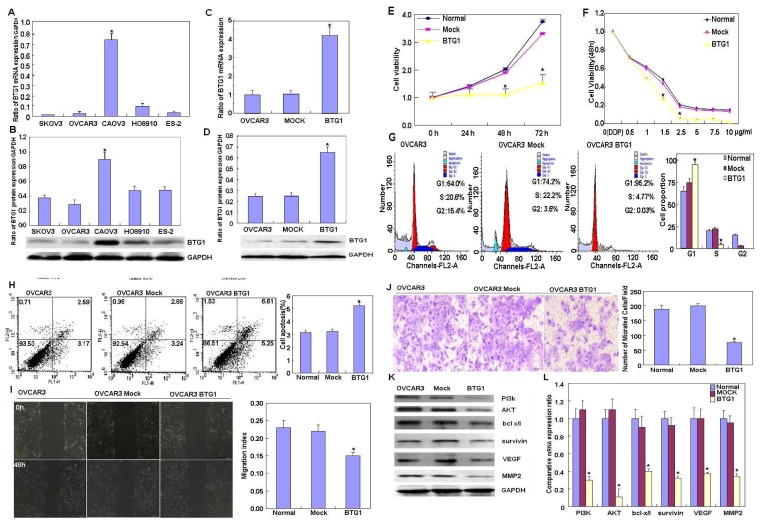
Effects of *BTG1* overexpression on the phenotypes and related proteins of ovarian carcinoma cells. *BTG1* mRNA and protein expression was screened in ovarian carcinoma cells (OVCAR3, SKOV3, HO8910, ES-2, and CAOV3); *BTG1* mRNA (**A**) and protein (**B**) expression in CAOV3 cells was higher as compared with the other cell lines; After transfection with *BTG1*-expressing plasmids, *BTG1* overexpression was detected in OVCAR3 cells by real-time RT-PCR (**C**) and western blotting (**D**) as compared with control (CTR) cells. The transfectants had lower growth and (**E**) and were sensitive to cisplatin treatment: cell viability decreased significantly at 1.5 μg/mL and 2.5 μg/mL cisplatin (**F**); and there was G_1_ arrest (**G**); a higher rate of apoptosis (**H**); less migration (**I**); and slower invasion (**J**) as compared to CTR cells. After forced *BTG1* overexpression, there was reduced *PI3K*, *Akt*, *Bcl-xL*, *survivin*, *VEGF*, and *MMP-2* mRNA (**K**) and protein expression (**L**) in OVCAR3 cells. *p < 0.05. Results are representative of three separate experiments; data are expressed as the mean ± standard deviation, with CTR as “1”.

Real-time RT-PCR and western blotting revealed that *BTG1* mRNA and protein expression, respectively, were higher in CAOV3 cells as compared with other carcinoma cells (Figure 1A,B, *p* < 0.05). To clarify the role of *BTG1*, a *BTG1*-expressing plasmid was transfected into OVCAR3 cells, as shown by real-time PCR (Figure 1C, *p* < 0.05) and western blotting (Figure 1D, *p* < 0.05). In comparison with the negative control and mock cells, Cell Counting Kit-8 (Dojindo, Kumamoto, Japan) revealed that the transfectants had a lower rate of growth (Figure 1E, *p* < 0.05) and higher cisplatin sensitivity (Figure 1F, *p* > 0.05); propidium iodide (PI) staining revealed that there was G_1_ arrest (Figure 1G, *p* < 0.05). Annexin V-fluorescein isothiocyanate (FITC) staining revealed the apoptosis-inducing effect of *BTG1* overexpression in OVCAR3 cells (Figure 1H, *p* < 0.05). Wound healing and Transwell assays revealed slower migration (Figure 1I, *p* < 0.05) and invasion (Figure 1J, *p* < 0.05), respectively, by transfectants as compared to the negative control and mock cells. Additionally, there was lower phosphoinositide 3-kinase (PI3K), protein kinase B (Akt) Bcl-xL, survivin, vascular endothelial growth factor (VEGF), and matrix metalloproteinase-2 (MMP-2) protein (Figure 1K) and mRNA (Figure 1L) expression in the *BTG1* transfectants as compared with the negative control and mock cells.

### 2.2. Correlation of *BTG1* mRNA Expression with Tumorigenesis and Clinicopathological Features of Ovarian Carcinoma

*BTG1* mRNA in ovarian tissue was amplified using real-time RT-PCR. There was higher expression of *BTG1* mRNA in normal tissue than in carcinoma tissue (*p* = 0.001, Table 1) and in benign tumors than in carcinomas (*p* = 0.027, Table 1), as well as in International Federation of Gynecology and Obstetrics (FIGO) stage I/II carcinomas than in FIGO stage III/IV carcinomas (*p* = 0.038; Table 2). *BTG1* mRNA expression was not correlated with pathological classification or differentiation of ovarian carcinoma (*p* > 0.05; Table 2).

## 3. Discussion

An increasing amount of evidence suggests that *BTG1* is a member of a family of antiproliferative genes, as *BTG1* expression is highest in the G_0_/G_1_ phases of the cell cycle and is downregulated when cells progress through G_1_[4,11]. The present study is the first instance that *BTG1* mRNA expression in precancerous and cancerous ovarian tissue was examined. *BTG1* mRNA expression was lower in carcinomas and higher in normal ovarian tissue, indicating that downregulated *BTG1* expression contributes to ovarian epithelial carcinogenesis. Our findings are in agreement with the finding that *BTG3* mRNA expression was higher in ovarian normal tissue and benign tumors than that in borderline, primary, and metastatic ovarian carcinoma [21]. Urzúa *et al*. [22] used cDNA microarray analysis and demonstrated a significant association between aberrant *BTG1* mRNA expression and ovarian carcinoma. *BTG1* downregulation was reported to result from genomic deletions, which were detected in 9% (65/722) of B-cell precursor acute lymphoblastic leukemia (ALL), but not in 109 cases of T-cell ALL [23]. In future studies, we aim to clarify the expression and genetic/epigenetic alteration of *BTG1*.

In the present study, *BTG1* mRNA expression was negatively linked to FIGO staging of ovarian carcinoma, indicating that BTG1 protein might be involved in the development of ovarian cancer and may be considered a good biomarker for indicating the aggressive behaviors of ovarian carcinoma. Deng *et al*. [24] found that *BTG3* mRNA expression was negatively correlated with dedifferentiation and FIGO staging of ovarian carcinoma. Our data are in accordance with that of Chen *et al*. [25], who reported that *BTG1* expression was higher in the prostate carcinoma cell line LNCaP than in the aggressively metastatic AI C4-2 cell line at both mRNA and protein level. Wang *et al*. [26] treated LNCaP cells with the androgen receptor antagonist flutamide and found that *BTG1* mRNA expression was upregulated, indicating that *BTG1* overexpression might be involved in the enhancement of chemotherapeutic sensitivity in cancers.

After investigating the *BTG1* mRNA and protein expression in all cell lines, we found that *BTG1* mRNA and protein expression were higher in CAOV3 cells than in the other cell lines (*p* < 0.05); there were no significant differences between the other cell lines. We used the OVCAR3 cell line for *BTG1* transfection because of its higher proliferation ability. In agreement with its function as a tumor suppressor gene, our *in vitro* study showed that ectopic *BTG1* overexpression inhibited proliferation, migration, and invasion and induced G_1_ arrest and apoptosis in OVCAR3 cells. Forced *BTG1* overexpression decreased the expression of *PI3K*, *Akt*, *Bcl-xL*, *survivin*, *VEGF*, and *MMP-2* mRNA and protein; these are target factors in our cancer research, which have clear roles in the regulation of cellular apoptosis and proliferation, and thus were examined in this study. Other proteins, such as Wnt-5a, Notch, MDR and GST-π, were also analyzed, but did not yield significant results.

## 4. Materials and Methods

### 4.1. Cell Culture and Transfection

Ovarian carcinoma cell lines CAOV3 (serous adenocarcinoma), OVCAR3 (serous cystic adenocarcinoma), SKOV3 (serous papillary cystic adenocarcinoma), HO8910 (serous cystic adenocarcinoma), and ES-2 (clear cell carcinoma) were purchased from ATCC (Manassas, VA, USA). They were maintained in RPMI 1640 (ES-2, HO8910, and OVCAR3), DMEM (CAOV3), and McCoy’s 5A (SKOV3) medium supplemented with 10% fetal bovine serum (FBS), 100 units/mL penicillin, and 100 μg/mL streptomycin in a humidified atmosphere of 5% CO_2_ at 37 °C.

### 4.2. Plasmid Construction and Transfection

*BTG1* was amplified using the following primer sequences: forward, 5′-CCGGAATTCATGCATCCCTTCTACACC-3′; reverse, 5′-GCTCTAGAACCTGATACAGTCATCATAT-3′. Template cDNA was obtained from human colorectal carcinoma HCT115 cells. PCR products were ligated into a pcDNA3.1 vector (Clontech, Mountain View, CA, USA) between the *Eco*RI and *Xba*I sites.

OVCAR3 cells were transfected with pcDNA3.1-*BTG1* or empty pcDNA3.1 24 h after they were seeded on dishes and selected by G418 according to the manufacturer’s instructions (Qiagen, Valencia, CA, USA); the final collection comprised three monoclones, which were combined for the following experiments.

### 4.3. Cell Cycle Analysis

The cells were trypsinized, collected, washed with phosphate-buffered saline (PBS) twice, and fixed in 10 mL cold ethanol for 12 h. Then, the cells were washed with PBS twice and incubated with 1 mL RNase (0.25 mg/mL) at 37 °C for 1 h before they were pelleted and resuspended in 50 μg/mL PI and incubated at 4 °C in the dark for 30 min. The PI signal was detected using flow cytometry.

### 4.4. Apoptosis Assay

To detect phosphatidylserine externalization as an endpoint indicator of early apoptosis, flow cytometry was performed using 7-amino actinomycin and FITC-labeled annexin V (CA92121; BD Biosciences, San Diego, CA, USA) according to the manufacturer’s instructions.

### 4.5. Wound Healing Assay

Cells (1.0 × 10^6^ cells/well) were seeded in 6-well culture plates. After they had grown to confluence, the monolayer was scraped with a pipette tip (200 μL) into nine areas to create a scratch, washed with PBS thrice, and cultured in FBS-free medium. Cells were photographed at 0, 12, 24, and 48 h (*n* = 9) and the scratch area was measured using Scion Image for Windows (NIH, Bethesda, MD, USA). The wound healing rate = (area of original wound − area of actual wound at different times)/area of original wound × 100%.

### 4.6. Cell Invasion Assays

A thin Matrigel layer (40 μL of 8 mg/mL stock solution; Becton Dickinson, Bedford, MA, USA) was overlaid on the top chamber of 6.5-mm Transwell plates (8-μm pore size; BD Biosciences, 354481). The Matrigel was allowed to solidify by incubating the plates for 4 h at 37 °C. Culture medium was added to the bottom chamber of the Transwell plates. Stable clones for *BTG1* (*n* = 3), vector control (*n* = 3), and negative control (*n* = 3) were resuspended in serum-free RPMI 1640 at a concentration of 2.5 × 10^5^ cells/mL, and 5 × 10^4^ cells were added to the top chamber of the Transwell plate. Following 48-h incubation, cells that had not invaded through the Matrigel were removed from the top surface using cotton swabs. Cells that had invaded through the Matrigel and reached the bottom surface of the filters were fixed in methanol and stained with 0.1% crystal violet. Invasion was quantified by counting the number of cells under an Olympus fluorescence microscope equipped with a 16-square reticle; the surface area of this grid was 1 mm^2^. Five separate fields were counted for each filter and the total numbers of cells were compared among groups using the Student *t*-test with the assumption of two-tailed distribution and two samples with equal variance. A difference of *p* < 0.05 was deemed statistically significant.

### 4.7. Subjects

Between January 2003 and December 2011, ovarian normal tissue (*n* = 17), benign tumors (*n* = 12), and carcinoma specimens (*n* = 64) from surgical resection were collected from the Department of Obstetrics and Gynecology, The First Hospital Affiliated to China Medical University, Shenyang, China. The average age at surgery was 50.8 years (range 20–80 years). Specimens were frozen immediately in liquid nitrogen and stored at −80 °C until used. None of the patients had undergone chemotherapy, radiotherapy, or adjuvant treatment before surgery. Informed consent was obtained from all subjects and the China Medical University Ethics Committee approved the study.

### 4.8. Pathology

All specimens were fixed in 10% neutral formalin, embedded in paraffin, and 4-μm sections were obtained. Sections were stained with hematoxylin-eosin to confirm their histological diagnosis and other microscopic characteristics. Each ovarian carcinoma specimen was evaluated according to the FIGO staging system. The histology of the ovarian carcinoma specimens was described according to World Health Organization classification.

### 4.9. Real-Time RT-PCR

Total RNA was extracted from the specimens using an RNeasy mini kit (Qiagen, Hilden, Germany). Total RNA (2 μg) underwent cDNA synthesis using avian myeloblastosis virus transcriptase and random primers (Takara, Otsu, Japan; Table S1). PCR amplification of cDNA was performed in 20-μL reactions according to the SYBR Premix Ex Taq^™^ II kit protocol (Takara, Otsu, Japan).

### 4.10. Western Blotting

Denatured proteins were separated on a sodium dodecyl sulfate-polyacrylamide gel (12% acrylamide) and transferred to a Hybond membrane (Amersham, Munich, Germany), which was blocked overnight with 5% skimmed milk in TBST (10 mmol/L Tris-HCl, 150 mmol/L NaCl, 0.1% Tween 20). For immunoblotting, the membrane was incubated for 1 h with primary antibodies against BTG1 (1:1000; Proteintech, Chicago, IL, USA), PI3K, Akt, Bcl-xL, survivin, VEGF, and MMP-2 (Santa Cruz Biotechnology, Santa Cruz, CA, USA). Then, it was rinsed with TBST and incubated with horseradish peroxidase–conjugated IgG (1:1000; Dako, Carpinteria, CA, USA) for 1 h. Bands were visualized with X film (Fujifilm, Tokyo, Japan) using ECL Plus detection reagents (Santa Cruz Biotechnology, Santa Cruz, CA, USA). Subsequently, the membrane was washed with WB Stripping Solution (pH 2–3; Nacalai Tesque, Tokyo, Japan) for 1 h and treated as described above except that anti-GAPDH antibody (1:1000; Santa Cruz Biotechnology, Santa Cruz, CA, USA) was used as an internal control. Densitometric quantification of the target proteins was performed with a GAPDH control using Scion Image (Version 4.0.3.2, Scion Corporation, Frederick, MD, USA, 2008) for Windows.

### 4.11. Statistical Analysis

Statistical evaluation was performed using Spearman’s correlation test and the Wilcoxon test to analyze the rank data and to differentiate the means of different groups, respectively. SPSS 10.0 (IBM, Armonk, NY, USA, 1999) was used to analyze all data and *p* < 0.05 was deemed statistically significant.

## 5. Conclusions

In summary, our findings suggest that upregulated *BTG1* expression might suppress the aggressive phenotypes of ovarian carcinoma cells by downregulating expression of the phenotype-related genes and subsequently their encoded protein products, such as members of the PI3K-Akt pathway, Bcl-xL, survivin; VEGF; and MMP-2 which promote proliferation, anti-apoptosis, and invasion, respectively [27–29]. Our study indicates that altered *BTG1* expression might affect ovarian carcinogenesis and may be considered a potential biomarker for ovarian carcinogenesis and progression by modulating proliferative, apoptotic, migratory, and invasive events. Nevertheless, the biological functions of *BTG1* in ovarian carcinoma require further investigation.

## Supplementary Information



## Figures and Tables

**Table 1:** *BTG1* mRNA expression in ovarian epithelial carcinogenesis.

Group	*n*	*BTG1* mRNA expression
Normal	17	0.197 ± 0.080 **
Benign tumors	12	0.127 ± 0.077 *
Carcinoma	64	0.077 ± 0.059

** Compared with normal tissue (*p* = 0.001);

* compared with benign tumors (*p* = 0.027).

**Table 2:** Correlation of *BTG1* mRNA expression with tumorigenesis and aggressive features of ovarian carcinoma.

Clinicopathological features	*n*	*BTG1* mRNA expression
**Pathological classification**		
Serous adenocarcinoma	50	0.076 ± 0.062
Mucinous adenocarcinoma	7	0.069 ± 0.038
Miscellaneous subtypes	7	0.089 ± 0.059
FIGO staging:		
I–II	30	0.093 ± 0.069 *
III–IV	34	0.063 ± 0.045
**Differentiation**		
Well-differentiated	21	0.084 ± 0.046
Moderately differentiated	22	0.076 ± 0.053
Poorly differentiated	21	0.071 ± 0.076

* Compared with International Federation of Gynecology and Obstetrics (FIGO) stage I/II (*p* = 0.038).
