# Supplementary material for: BTG1 Expression Correlates with the Pathogenesis and Progression of Ovarian Carcinomas
Source: Int J Mol Sci. 2013 Sep 27;14(10):19670–80. doi: 10.3390/ijms141019670 (PMC3821579; doi:10.3390/ijms141019670)
Supplement: Supplementary file 1 [file ijms-14-19670-s001.pdf]

# Supplementary Information

**Table S1.** Primers used for RT-PCR.

| Primer | Sequences (forward and reverse) | Distribution | AT (°C) | Product size (bp) | Extension time (s) |
|---|---|---|---|---|---|
| *BTG1* | 5'-CCCTTCCTGGGCATCGT-3'<br>5'-CGGCGTCGTCATCATCC-3' | L10240.1<br>645–741 | 60 | 97 | 34 |
| *PI3K* | 5'-CACCGCATTTGTCGT-3'<br>5'-CTCCCACTTCTACGC-3' | NM_001242466<br>4016–4240 | 60 | 225 | 34 |
| *Akt* | 5'-GGGACAGAGGAGCAAGGT-3'<br>5'-CGACAGCGGAAAGGTTAA-3' | NM_005163<br>2656–2863 | 60 | 208 | 34 |
| *Bcl xl* | 5'-TTCCCAGAAAGGATACAGC-3'<br>5'-GGGTCTCCATCTCCGATT-3′ | Z23115<br>185–276 | 60 | 92 | 34 |
| *survivin* | 5'-CTTGGCCCAGTGTTTCTT-3'<br>5'-GCTTCCAGTCCCTCCCT-3' | DQ227257<br>159–282 | 60 | 124 | 34 |
| *VEGF* | 5'-AGGAGGAGGGCAGAATC-3′<br>5'-ATGTGCTGGCCTTGGT-3' | NM_001171630<br>1131–1388 | 60 | 258 | 34 |
| *MMP-2* | 5'-GAGCACTCCCAAGACCCT-3'<br>5'-AGTCCGCCAAATGAACC-3' | NM_001127891<br>1398–1652 | 60 | 255 | 34 |
| *GAPDH* | 5'-CAATGACCCCTTCATTGACC-3'<br>5'-TGGAAGATGGTGATGGGATT-3' | NM_002046.3<br>201–335 | 60 | 135 | 34 |

AT, annealing temperature; *GADPH*, glyceraldehyde-3-phosphate dehydrogenase.

© 2013 by the authors; licensee MDPI, Basel, Switzerland. This article is an open access article distributed under the terms and conditions of the Creative Commons Attribution license (http://creativecommons.org/licenses/by/3.0/).
